# *Clostridium novyi*-NT can cause regression of orthotopically implanted glioblastomas in rats

**DOI:** 10.18632/oncotarget.3627

**Published:** 2015-03-18

**Authors:** Verena Staedtke, Ren-Yuan Bai, Weiyun Sun, Judy Huang, Kathleen Kazuko Kibler, Betty M. Tyler, Gary L. Gallia, Kenneth Kinzler, Bert Vogelstein, Shibin Zhou, Gregory J. Riggins

**Affiliations:** ^1^ Department of Neurology & Neurosurgery, Sidney Kimmel Cancer Center, Johns Hopkins University School of Medicine, Baltimore, MD, USA; ^2^ Department of Oncology, Sidney Kimmel Cancer Center, Johns Hopkins University School of Medicine, Baltimore, MD, USA; ^3^ Department of Pediatrics-Anesthesiology, Baylor College of Medicine, Houston, TX, USA

**Keywords:** *Clostridium novyi*-NT, bacterial therapy, glioblastoma multiforme, stroke

## Abstract

Glioblastoma (GBM) is a highly aggressive primary brain tumor that is especially difficult to treat. The tumor's ability to withstand hypoxia leads to enhanced cancer cell survival and therapy resistance, but also yields a microenvironment that is in many aspects unique within the human body, thus offering potential therapeutic opportunities. The spore-forming anaerobic bacterium *Clostridium novyi-*NT(*C. novyi-*NT) has the ability to propagate in tumor-generated hypoxia, leading to oncolysis. Here, we show that intravenously injected spores of *C. novyi-*NT led to dramatic tumor destructions and significant survival increases in implanted, intracranial syngeneic F98 and human xenograft 060919 rat GBM models. *C. novyi*-NT germination was specific and confined to the neoplasm, with sparing of the normal brain parenchyma. All animals tolerated the bacteriolytic treatment, but edema and increased intracranial pressure could quickly be lethal if not monitored and medically managed with hydration and antibiotics. These results provide pre-clinical data supporting the development of this therapeutic approach for the treatment of patients with GBM.

## INTRODUCTION

Approximately 3% of all cancer deaths are attributed to primary brain tumors [1]. Among those, glioblastoma multiforme (GBM), a grade IV astrocytoma arising from the glial tissue, is the most common (65%) and deadly primary brain malignancy [1]. As the “multiforme” component implies, GBM exhibits large variability at the histopathological level, with extensive necrotic regions intermixed with cell proliferation and infiltration [2]. GBMs are one of the most difficult cancer types to treat, with a 5-year survival rate of less than 5% [1]. This is so despite >600 clinical trials and multi-mode treatments that include surgical resection, local delivery of BCNU (Gliadel wafers), and concurrent chemoradiotherapy with temozolomide (TMZ) followed by adjuvant TMZ. Reasons for treatment failure are complex and include incomplete surgical excision due to the tumor's invasiveness as well as insufficient drug delivery to the remaining tumor cells [3].

Unlike hematological cancers, most solid tumors and in particular GBM rapidly outgrow their vascular supply and develop tumor hypoxia, complicating treatment with traditional cancer therapies [4]. The poor tumor vascular supply inevitably limits the delivery of systemically administered chemotherapeutic drugs and reduces the effectiveness of radiotherapy [5]. Tumor hypoxia has a central role in tumor metabolism, progression, metastasis, and immune evasion [4]. Though conceptually a promising target, a means to successfully utilize tumor hypoxia has not yet been achieved. Previous efforts focusing on molecular targeting with small molecules has had only limited success [6].

Another approach to exploit the hypoxic nature of the tumor environment involves anaerobic bacteria. The oncolytic ability of bacteria has been recognized since the early 1800s, as exemplified by the development of Coley's vaccine [7]. Various species of *Clostridium*, *Salmonella*, *Escherichia*, *Bifidobacterium* and *Listeria* have all been considered for this purpose [8]. One more recently recognized species is *Clostridium novyi*, which in its therapeutic form is devoid of the lethal alpha toxin gene, termed *C. novyi*-NT [9]. *C. novyi* is a highly mobile, spore-forming bacterium that is exquisitely sensitive to oxygen. As an obligate anerobe, vegetative forms cannot survive in oxygen and bacterial spores can only germinate in hypoxic conditions [10]. Previous studies showed that a single dose of *C. novyi*-NT spores injected intravenously in syngeneic tumor-bearing animals often led to localized tumor necrosis and oncolysis, leading to cures in up to one-third of treated animals, without excessive toxicity [11, 12].

In this work we show that treatment of GBM-bearing animals with *C. novyi*-NT spores results in localized germination, tumor destruction and a significant survival benefit. These encouraging results suggest that the approach should be developed further.

## RESULTS

### I.V. injected *C. novyi*-NT spores increase survival in rodent glioblastoma models

**Figure 1:**
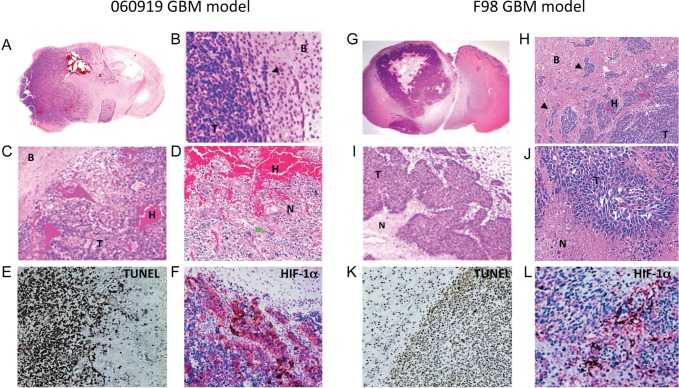
Histological characterization of the intracranial human xenograft 060919 and syngeneic rat F98 GBM models 060919 and F98 GBM were collected, paraffin-embedded and sectioned for H&E, TUNEL and HIF-1α staining at 28 and 18 days after tumor implantation respectively. (A-D) Anatomical view and histology by H&E of 060919 GBM. Tumors are characterized by a highly invasive growth pattern invasion (black arrowhead; A, B), intratumoral hemorrhages (C), giant cells (green arrowhead; C) and necrotic regions intermixed with cell proliferation (D), closely resembling human disease. (E) TUNEL staining of 060919 GBM. (F) HIF-α staining of 060919 GBM. (G-J) Anatomical view and histology by H&E of syngeneic F98 GBM. Similar to the human 060919 xenograft, syngeneic F98 GBM are highly infiltrative (black arrowhead; G, H) with intratumoral hemorrhages (H), extensive areas of necrosis (I, J) and pseudopalisidation (J). (K) TUNEL staining of F98 GBM. (L) HIF-α staining of F98 GBM. Abbreviations: T: Tumor. N: Necrosis. H: Hemorrhage. B: Brain. Black arrowhead: invasions. Green arrowhead: giant cells.

To assess the potential value of *C. novyi*-NT bacteriolytic therapy for intracranial malignancies, we used two different rodent orthotopic GBM models. Both models were highly invasive and known to form intratumoral necrosis as well as hypoxia in rodents (Fig. 1A-L), characteristics found in their human counterparts.

**Figure 2:**
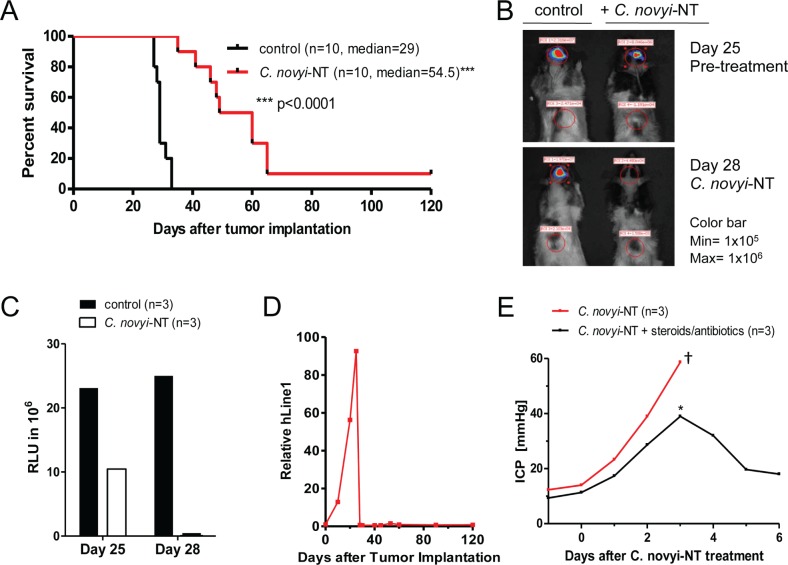
*C. novyi*-NT increases survival in intracranial 060919 GBM xenograft model (A) Kaplan-Meier curve of 060919 xenografts treated with a single dose of 3×10^8^ IV injected *C. novyi*-NT spores at day 25. *C. novyi*-NT treatment increased survival by 26 days (a 90% increase) and for one animal treatment resulted in long-term survival. (B) Athymic rats implanted with 060919 cells expressing firefly luciferase were injected with 50 mg/kg luciferin and evaluated *via* Xenogen before and after the treatment with *C. novyi*-NT spores. Clostridial treatment led to a dramatic reduction in the luciferase signal (>90%) in the first 48 hours, which along with the h-LINE1 served as an indicator for a favorable treatment response. (C) Total luciferase counts of *C. novyi*-NT-treated and untreated animals are displayed. (D) Tumor burden was monitored *via* h-LINE1 content in the peripheral blood. A decrease in h-LINE1 or absence thereof correlated with decreased tumor burden and subsequent increased survival. (E) Intracranial 060919 glioblastoma-bearing athymic rats were monitored daily for changes in the intracranial pressure during bacteriolytic therapy using an intraventricular ICP measurement device. During bacteriolytic treatment ICP increased significantly and required medical treatment with steroids (10 mg/kg) and antibiotics (doxycycline 50 mg/kg). Animals with ICPs of ≤ 40 mmHg (*) responded well to medical management with a prompt ICP reduction and recovery, whereas animals with ICP values of >45 mmHg died (†)

The GBM neurosphere cell line 060919 was grown from a surgically resected GBM in serum-free media [13]. 060916 cells were implanted intracranially and allowed to grown until day 25 when the rats were injected with 3×10^8^*C. novyi*-NT spores *via* tail vein. Therapy with *C. novyi-*NT resulted in a significant overall survival improvement and one long-term surviving rat of 10 that were treated (Fig. 2A). Bacterial germination began within 12 hours after injection and was florid by 24 hours, as evidenced by the presence of many vegetative bacteria on histological sections of the tumor. Successful tumor lysis was evident by the dramatic decline in luciferase signal (>90%) in the first 48 hours and the rapid reduction of circulating human DNA, both of which served as indicators of tumor burden (Fig. 2B-D).

Commonly observed side effects of bacteriolytic therapy included lethargy, brain edema and increased intracranial pressure (ICP). We found that increased ICP was a major cause of death if not detected early and treated aggressively with steroids and antibiotics. Animals with ICPs ≤ 40 mmHg readily responded to medical management, measured by an immediate ICP reduction, and fully recovered (Fig. 2E). Animals with ICPs of >45 mmHg did not respond to medical therapy and subsequently died.

**Figure 3:**
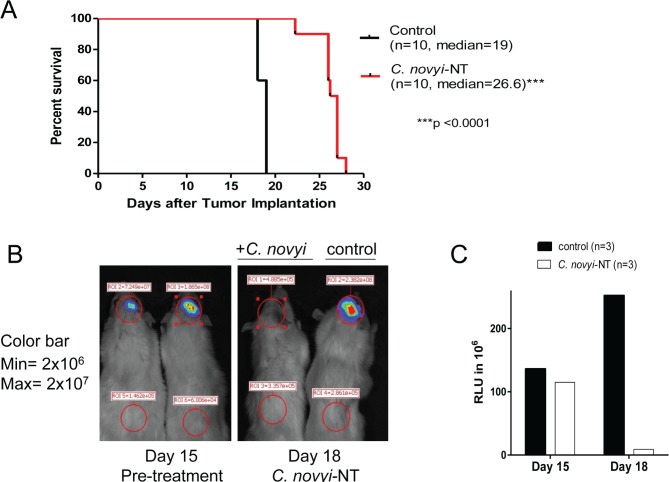
*C. novyi*-NT increases survival in syngeneic intracranial F98 GBM model (A) Kaplan-Meier curve of syngeneic F98 GBM bearing Fisher rats treated with a single dose of 3×10^8^ IV injected *C. novyi*-NT spores at day 15. Bacterial treatment led to a 42% survival extension. (B) Fisher rats implanted with F98 cells expressing firefly luciferase were injected with 50mg/kg luciferin and evaluated *via* Xenogen before and after the treatment with *C. novyi*-NT spores. Similar to the results for 060919, we found a >90% reduction in the luciferase signal, an indicator for successful tumor lysis by *C. novyi*-NT. (C) Total luciferase counts of *C. novyi-*NT-treated and untreated animals are displayed

To determine whether these results could be reproduced in an immune-competent rodent model, we used F98 cells that were originally produced by IV administration of N-ethyl-N-nitrosourea, a highly potent mutagen, into pregnant Fisher F344 rats that are refractory to a number of chemotherapeutics. Treatment of this aggressive intracerebral tumor with *C. novyi*-NT significantly prolonged survival (Fig. 3A). Similar to the 060919 model, a dramatic reduction in luciferase activity was present 48 hours after treatment compared to the untreated controls (Fig. 3B, C). However, tumor eradication was incomplete, particularly in the less hypoxic tumor rim, and tumors eventually regrew.

**Figure 4:**
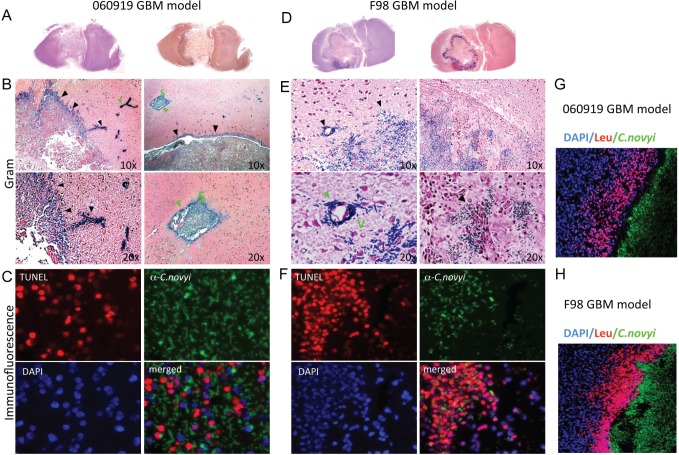
Micrographs of tumoral *C. novyi*-NT germination in human 060919 and syngeneic F98 GBM models (A-C) Anatomical view and histology of 060919 GBM treated with *C. novyi*-NT. (B) Gram stained brain sections revealed that vegetative *C. novyi*–NT bacteria are precisely confined to the tumor (black arrowhead) and in satellite microinvasions of neoplastic cells (green arrowhead) but not in normal brain parenchyma. (C) Immunofluorescence staining for DAPI stained nuclei blue, TUNEL (red) and vegetative *C. novyi*-NT (green) in 060919 bearing rats were evaluated by confocal laser microscopy. *C. novyi*-NT co-localized with TUNEL positive tumor cells. (D-F) Anatomical view and histology of syngeneic F98 GBM treated with *C. novyi*-NT. (E) Similarly to 060919, vegetative *C. novyi*–NT bacteria are exclusively localized to the tumor (black arrowhead) as well as neoplastic vessels (green arrowhead). (F) *C. novyi* antibody stained vegetative bacteria were visualized in TUNEL positive tumor regions, but not in that of TUNEL negative cells. (G, H) *C. novyi*-NT induces a potent local host-inflammatory response in 060919 (G) and F98 (H) bearing animal. Brains were examined 24-48h after systemic injection of *C. novyi*-NT. A ring of leukocytes, stained by CD45, surrounded the tumor and restrained the infection (*C. novyi*-NT in green). DAPI is shown in blue. Abbreviations: S: Satellite microinvasions. V: Neoplastic vessel

Histological analysis of brain sections of both high-grade glioma models revealed that bacterial germination was precisely localized to the tumor and involved the tumor body as well as distant tumor satellites, while the normal brain parenchyma was unaffected (Fig. 4A, B, D, E). Within tumors, vegetative bacterial distribution was not random; it was strictly co-localized with TUNEL positive tumor cells (red) (Fig. 4C, F). Importantly, germination was not limited to the larger tumor mass and extended to stem-like protrusions from the main tumor, as well as microscopic distant tumor satellites and tumor vessels (Fig. 4B, E). Bacterial infection was accompanied by a striking accumulation of host inflammatory cells, predominantly neutrophils at the periphery, presumably limiting bacterial spread (Fig. 4G, H).

### Combination with liposomal doxorubicin has synergistic effects

**Figure 5:**
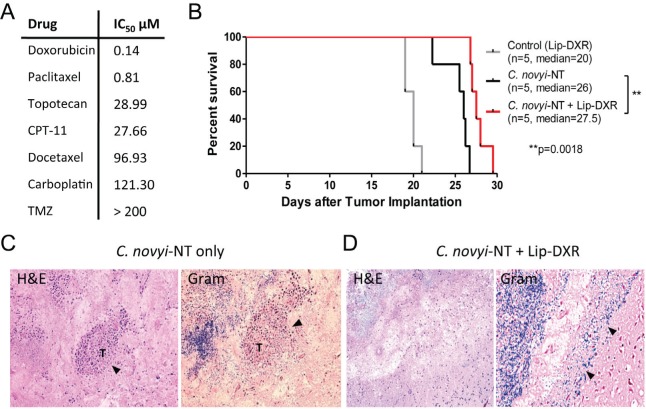
Combination of *C. novyi-*NT with liposomal doxorubicin improves the clearance of the tumor rim in syngeneic F98 GBM model (A) The half-maximal inhibitory concentrations (IC_50_) levels of seven chemotherapeutics including temozolomide (TMZ) in F98 cells are shown. Doxorubicin appears to be superior with an IC_50_ of 0.14 μM. (B) Kaplan-Meier survival curve of F98 GBM treated with a combination of Lip-DXR (5 mg/kg) and *C. novyi*-NT spores. The addition of Lip-DXR extended the survival by 10% compared to *C. novyi*-NT alone. Lip-DXR alone had no effect on the survival. (C, D) H&E and gram staining of the brain sections. Histologic examination revealed that rats undergoing combination therapy with *C. novyi*-NT spores plus Lip-DXR had lower amounts of remaining viable tumor seen on H&E sections (D) when compared to *C. novyi*-NT alone where larger portions of viable tumor (T) were noted, particularly in the periphery as indicated in the sections (C)

Though the effect of *C. novyi*-NT treatment is dramatic, long-term survival is rarely achieved and hence, a combination with other complementary chemotherapeutic agents could prove useful. For this purpose, we first determined the half-maximal inhibitory concentrations (IC_50_) of seven commonly used chemotherapeutic agents of F98 cells *in vitro*. The most potent proved to be doxorubicin, a DNA intercalating substance, with an IC_50_ of 0.14μM (Fig. 5A). A liposomal formulation of doxorubicin (Lip-DXR) has previously been reported to enhance the anti-tumor efficacy of *C. novyi*-NT and may act as a depot for sustainable drug release [14]. In the syngeneic F98 GBM model, the combination of Lip-DXR and *C. novyi*-NT increased the survival modestly compared to *C. novyi*-NT alone (Fig. 5B). Tumors treated with *C. novyi*-NT spores plus Lip-DXR had improved tumor clearance, as exemplified by the lower number of viable tumor cells, particularly in the periphery (Fig. 5C, D).

### *C. novyi*-NT germination is specific to tumor generated hypoxia and necrosis

**Figure 6:**
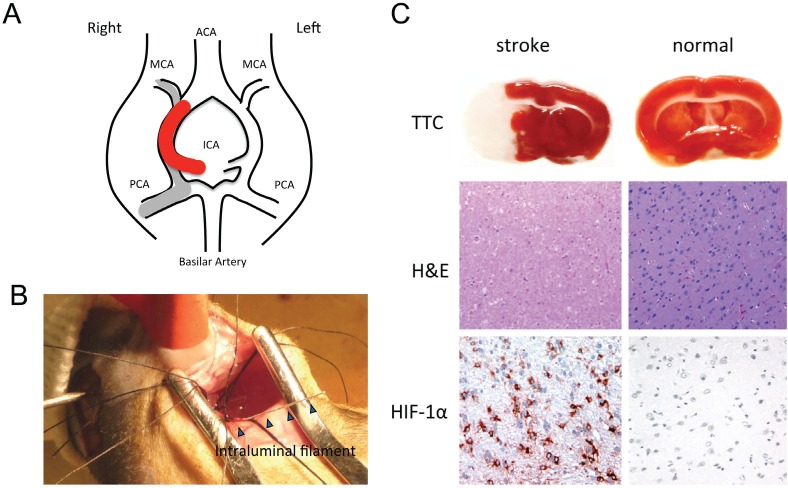
The intraluminal filament rat MCAO stroke model (A) Structural diagram of the vascular structures in the rat brain. The monofilament was inserted into the internal carotid artery (ICA) and advanced to the origin of the MCA, as indicated by the thick red line. This resulted in an ischemic infarction in the MCA territory (grey line). (B) Photograph taking during MCAO microsurgery showing the placement of the 4-0 nylon filament. (C) Representative coronal brain slices were taken 24 h after focal ischemia. The ischemic brain is shown on the left: TTC-stained white color indicates the infarct lesion, and red color represents normal tissue. As a comparison, the normal brain (right) did not have any white regions upon TTC staining. Following, H&E staining of the same brain illustrated the pattern of ischemic damage. There was massive immunoreactivity for HIF-1α, a marker for hypoxia, in cells of the infarcted region. There was no immunoreactivity to HIF-1α in normal brain tissue (right).

**Figure 7:**
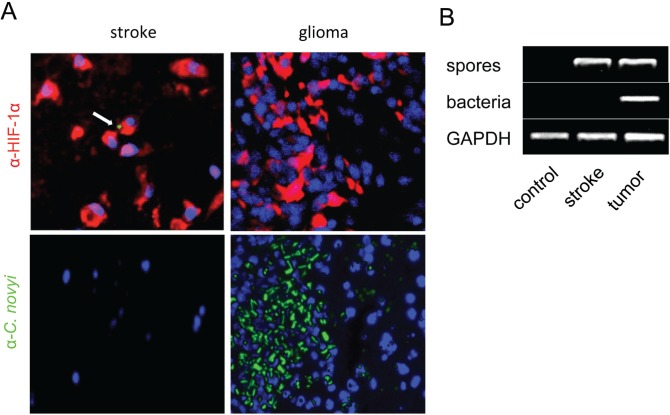
*C. novyi-*NT germinates in tumor hypoxia but not in non-malignant hypoxic tissue (A) Shown are sections of a brain with an ischemic stroke (left) and a F98 rat glioma (right). Both lesions, the stroke and brain tumor, have strong immunoreactivity for HIF-1α (red). However, despite the presence of *C. novyi*-NT spores (green, white arrow) in areas of the ischemic infarct, they did not germinate, as indicated by the absence of green staining. Spores in tumoral hypoxia were able to germinate (green) and co-localized with hypoxia-positive tumor cells. DAPI is shown in blue. (B) Consistent with the immunofluorescent data, PCR confirmed that vegetative bacteria were only present in the glioma but not in the infarct, although *C. novyi*-NT spores were found in both lesions

To determine if *C. novyi*-NT germination is specific to tumor associated hypoxia and necrosis, we established a cerebral ischemic rat middle cerebral artery occlusion stroke model. A large middle cerebral artery (MCA) infarct was created in normal athymic rats by transiently occluding the MCA while ligating the right internal carotid artery for 2 hours, thereby decreasing MCA blood flow by 70-90% (Fig. 6A, B). TTC and HIF-1α staining confirmed the infarction and hypoxia in the brain (Fig. 6C). Subsequently, 3×10^8^green-fluorescently labeled *C. novyi*-NT spores were injected into the tail vein and the animals observed for 5 days. Histopathological examination of the brains on day 5 demonstrated that, while the rats had large cerebral infarcts with extensive areas of necrosis, there was no histopathological or molecular-biologic evidence of bacterial colonization within or surrounding the lesions. *C. novyi-*NT spores (but not germinated bacteria) were found in the necrotic areas of the stroke, formally demonstrating that the absence of bacterial germination was not caused by the failure of spore delivery (Fig. 7A, B).

To prove that a sufficient degree of hypoxic injury was achieved in the stoke model, sections were stained with HIF-1α *via* immunofluorescence. HIF-1α is a key component of the cells' response to hypoxia, and the oxygen-labile HIF-1α protein is known to be induced in the central nervous system after focal ischemia caused by tumors or strokes [15]. The numbers of HIF-1α positive cells in the necrotic regions of tumors and strokes were similar, while bacterial germination was only evident in the tumors (Fig. 7A).

## DISCUSSION

Despite progress in the understanding, diagnosis and therapy of brain cancers, GBM remains a lethal disease. Complete surgical excision is nearly always impossible as a result of local infiltration and anatomical limitations, thus leading inevitably to tumor relapse [16]. Attempts to target common genetic defects in GBM have failed to provide long-term survival and have not replaced non-targeted approaches with chemotherapy and radiation [17]. Here, we investigated if spore-forming bacterium *C. novyi*-NT targeting the cancer's hypoxia might be technically feasible and beneficial in preclinical models of GBM.

Hypoxia is a critical aspect of glioblastoma biology and the development of resistance to chemotherapy and radiotherapy [4]. Though this hypoxic state represents a major therapeutic challenge, it also provides a potential opportunity. Anaerobic bacteria have evolved to thrive in the hypoxic environment and fortuitously some possess several key features that make them ideal anti-tumor agents [8]. The toxin-deleted strain *C. novyi-*NT appears to be particularly promising because: 1) bacteria germinate specifically in tumor-generated hypoxia but not in hypoxia generated by stroke (Fig. 6) or myocardial infarctions [12], (2) cytotoxicity is limited to the tumor while sparing adjacent normal tissues, and (3) vegetative *C. novyi*-NT are highly mobile, rapidly infecting the entire tumor. A single intravenous injection of *C. novyi-*NT spores into glioma-bearing rats resulted in florid germination *C. novyi*-NT's tumor-specificity is probably a result of the fact that hypoxia-driven necrosis in cancers may generate a steeper oxygen pressure gradient (dynamic hypoxia) compared to infarcted tissues (static hypoxia) with higher oxygen pressures [18].

Although the blood–brain barrier (BBB) normally protects the central nervous system from invading microorganisms, brain tumors or other inflammatory conditions can compromise its integrity and result in local breakdown of the BBB. This along with the fragile and leaky tumor vasculature manifesting in intratumoral hemorrhages can favor *C. novyi*-NT spore accumulation in the brain tumor bed. In addition, systemically administered *C. novyi*-NT spores could use the ‘Trojan horse’ method of entry by traveling in leukocytes. In this scenario *C. novyi*-NT spores are ingested by leukocytes where they remain dormant and once the infected leukocytes pass through the BBB, the spores escape as shown with *Clostridium difficile* [19].

With the advances in synthetic biology, there is a renewed interest in bacterial therapies for cancer [8]. Though the use of an attenuated strain of *C. novyi (*i.e., *C. novyi-NT*) reduces treatment-related toxicity, safety remains the major hurdle in clinical use [20]. In our study, the majority of rats treated with *C. novyi*-NT developed lethargy and signs of increased intracranial pressure due to local inflammation-driven edema that were medically manageable with the use of steroids and antibiotics, but the dangers of treating rats with intracranial tumors were evident. Similar observations were made by Diaz using tumor-bearing mice and rabbits, where edema and reversible inflammatory changes in the spleen, liver and adrenal glands were observed during spore treatment [12]. Most recently, Roberts reported a study in which ten pet dogs with spontaneous soft-tissue sarcomas treated with intratumoral injections of *C. novyi*-NT spores developed local edema and abscesses that required antibiotics or surgical excision of the infected tissues [21].

Truly effective cancer therapy will indubitably require a combination of multiple treatment modalities. Intravenous administration of *C. novyi*-NT enhances the effect of chemotherapy, particularly of hypoxia-enhancing agents, and radiation [11]. Consistent with those data, treatment with *C. novyi*-NT and liposomal doxorubicin resulted in a slightly increased survival benefit due to enhanced cytotoxicity [14]. It could also be valuable to combine *C. novyi*-NT with immune-checkpoint inhibitors. It has been demonstrated that long-term response to *C. novyi*-NT involves an immunologic T-cell based recognition that likely includes tumor antigens [22].

In summary, bacteriolytic therapy with the anaerobic spore forming bacterium *C. novyi*-NT is capable of precisely localizing to tumors and mediating robust tumoricidal activity with manageable side effects in two highly aggressive GBM models. The results provide a rationale for continuing to develop this approach for potential application in humans.

## MATERIALS AND METHODS

### Cell lines and tissue culture

Human GBM neurosphere line 060919 was grown in NeuroCult NS-A basal medium containing NeuroCult NS-A proliferation supplements (Stem Cell Technologies), 20 ng/mL epidermal growth factor (PeproTech), 10 ng/mL basic fibroblast growth factor (PeproTech), and 4 μg/mL heparin (Stem Cell Technologies). Rat F98 glioma cell line was maintained in Dulbecco's Modified Eagle Medium (DMEM) supplemented with 10% fetal bovine serum (FBS) and antibiotics. Both cell lines, 060910 and F98, were transfected with a luciferase construct *via* lentivirus (0609191-luc, F98-luc).

### Brain tumor models

6-week-old female F344 Fisher rats (weight 100-150 gram) and athymic nude rats were purchased from the National Cancer Institute (Bethesda, MD). For the implantation procedure, rats were anesthetized *via* intraperitoneal (i.p) injection composed of ketamine hydrochloride (75 mg/kg; 100 mg/mL; ketamine HCl; Abbot Laboratories), xylazine (7.5 mg/kg; 100 mg/mL; Xyla-ject; Phoenix Pharmaceutical), and ethanol (14.25%) in a sterile 0.9% NaCl solution. Following, 500,000 human GBM 060919 neurosphere cells infected with luciferase lentivirus were stereotactically implanted into the right frontal lobe located 3 mm lateral and 2 mm anterior to the bregma of athymic rats as previously described [23]. 20,000 F98-luc cells were stereotactically implanted into F344 Fisher rats using the same parameters. The tumors were allowed to grow until day 25 (060919) or 15 (F98), when 3×10^8^*C. novyi*-NT spores, produced as previously described [9], were injected into the tail vein. In addition, a subset of animals received liposomal doxorubicin (Doxil, Lip-DXR, Janssen, Titusville, NJ), 5 mg/kg i.p. at the time of spore injection. Pre- and post-treatment tumor sizes were assessed with a Xenogen instrument after intraperitoneal injection of 50mg/kg D-luciferin potassium salt per rat. During the first two days of bacterial therapy, rats were placed on 10 mg/kg/day dexamethasone i.p. to minimize the risk of postoperative edema. Control rats were stereotactically injected with the same volume of phosphate-buffered saline (PBS) and treated with dexamethasone (10 mg/kg per day) for the first 2 days. Animals were observed daily for any signs of deterioration, lethargy, neurotoxicity, or pain in accordance with the Johns Hopkins Animal Care and Use Guidelines. If symptoms of distress were present, supportive therapy with hydration and antibiotic metronidazole (loading dose of 15 mg/kg i.p. followed by 10 mg/kg every 12 hours as maintenance) was initiated and continued for a 7-day period. If symptoms persisted and/or resulted in debilitation, moribund animals were euthanized according to protocol. The anti-tumor efficacy of *C. novyi*-NT treatment was assessed with Kaplan-Meier survival curves, Xenogen results, and the degree of tumor burden on post-mortem brain sections. For the latter purpose, brains were harvested post-mortem, placed in formalin, and embedded in paraffin for additional pathological studies. Gram-stained slides, counter-stained with safranin, and H&E-slides were prepared according to routine histopathologic practices.

### Intracranial Pressure (ICP) measurement

Intracranial pressure was measured with an intraventricular catheter in athymic nude rats. In brief, a burrhole was drilled 1 mm posterior and 1 mm lateral to the bregma. Subsequently, a 2.5 mm long probe modified from a G20 needle by shortening the shaft and removing the plastic adaptor was inserted into the right lateral ventricle and glued to the skull. For measurements, a PE10 catheter was attached to the G20 needle, flashed with normal saline and connected with a Life Scope 6 pressure monitor (Nihon Kohden Corporation). The monitor was reset to zero before attaching to the probe.

### Stroke model

The intraluminal filament model of middle cerebral artery occlusion (MCAO) was used to induce focal ischemic injury to the brain in athymic nude rats [24]. Briefly, after anesthetization a laser-Doppler flow (LDF) probe was placed on the skull of the nude rat to monitor the perfusion. To induce a right-sided ischemic stroke, the right common carotid artery (CCA) was exposed through a submandibular midline incision. Then, the proximal end of the CCA was temporarily ligated and a 4-0 nylon monofilament was inserted into the internal carotid artery (ICA) and advanced past the CCA bifurcation to the origin of the MCA. After two hours, reperfusion of the right MCA was achieved by withdrawal of the filament and release of the CCA ligature. Animals were allowed to recover for 24 hours before 3×10^8^ FITC-fluorescent *C. novyi*-NT spores, produced as described by Diaz [12], were injected into the tail vein. Animals were sacrificed after 5 days. Post-mortem, brains were removed and incubated with 2,3,5-triphenyltetrazolium chloride (TTC) for 30 minutes to visualize the hypoxic areas as described [25]. Additionally, brains were embedded in paraffin for further histological analyses.

### Nested PCR for detection of vegetative forms of C. novyi-NT from brains

75 mg of fresh lesional brain tissue was harvested from sacrificed animals with strokes or GBM. The preparation of the tissue followed previous reports by Bettegowda [26]. RNA was prepared by homogenization of the dissected tissue in a glass homogenizer containing a ten-fold volume of ice-cold RNAwiz buffer (Ambion) followed by 12-cycle homogenization with Zirconia beads (Biospec) for 10 min at 4 °C to ensure disruption of the spores. RNA was isolated using the RNA Total Kit (Promega) and quantified with a UV spectrophotometer. First-strand cDNA was synthesized from total RNA using random oligonucleotides as primers and the SuperScript First-Strand Synthesis System (Invitrogen). Synthesized cDNA was then used for a nested PCR to detect *C. novyi*-NT's growth phase specific genes. The first run used primer pair (CCATAGCAATAAATCTTCCCTC and CATCTAAGCTTGAAACGTGTAG) targeting NT01CX2304, an indicator for vegetative bacteria, as well as primer pair (GCTAGTAAATGTGGATTTACTCC and ATTTCAGATGGTTCTGTGGTAG) targeting NT01CX2376, a marker for spores [16]. In the second run primer pair (CTCTTAATACCTCTTTCCCTTC and GTGTTTGTCATATGCTACTTCC) for NT01CX2304 and (CCTGGTAGTAACTCTGAAGTTA and GTGGTAGGTTCAAATCTACCAA) for NT01CX2376 were used. All PCR products were checked in 1.5% (wt/vol) agarose electrophoresis with 1× Tris-borate-EDTA (TBE) buffer, stained with ethidium bromide (0.2 μg/mL). DNA bands were detected under UV light.

### Human LINE-1 (hLINE-1) quantification

hLINE-1 was quantified in the blood from tumor-bearing athymic nude rats to monitor the tumor burden as described [27]. Briefly, approximately 50 μl of blood were collected at various time points and DNA was purified according to the instructions of the Qiagen DNA purification kit (Qiagen, Valencia, CA). Quantitative PCR employed 0.5 μL of 10 μmol/L forward primer FWD 5′-TCACTCAAAGCCGCTCAACTAC-3′ (IDT DNA; desalted, 25 nmol scale), 0.5 μL of 10 μmol/L reverse primer REV 5′-TCTGCCTTCATTTCGTTATGTACC-3′ (IDT DNA; desalted, 25 nmol scale) and the following cycling conditions (iCycler, Biorad): (94°C, 2 min) × 1, (94°C, 10 s; 67°C, 15 s; 70°C, 15 s) × 3, (94°C, 10 s; 64°C, 15 s; 70°C, 15 s) × 3, (94°C, 10 s; 61°C, 15 s; 70°C, 15 s) × 3, (94°C, 10 s; 59°C, 15 s; 70°C, 15 s) × 35.

### Histological staining

Post-mortem brains were fixed with 10% buffered formalin and paraffin-embedded. For histological analysis, sections were deparaffinized with xylene, and rehydrated in a graded ethanol series.

### TUNEL staining

TdT-mediated dUTP nick end labeling was performed using the DeadEnd™ TUNEL system, according to the manufacturer's instructions (Promega, Madison, WI).

### Immunohistochemistry/Immunofluorescence

Immunohistochemical and immunofluorescence experiments were carried out as previously described [28]. Briefly, non-specific binding sites were blocked by incubation with 10% goat serum in PBS for 1 h at room temperature. The following primary antibodies were used: HIF-1 alpha (Abcam), CD45 (Abcam), *C. novyi*-NT antibody [22]. Omission of the primary antibody served as the negative control. For immunohistochemical detection, HRP-linked anti-rabbit or anti-mouse secondary antibodies were used (EnVision™ kit, DAKO). Visualization with DAB was performed according to the manufacturer's instructions (DAKO, Carpinteria, CA). For immunofluorescence detection the following secondary antibodies were applied: anti-rabbit Alexa 488 or anti-mouse Alexa 594 (Invitrogen). Sections were counterstained with 4′,6-diamidino-2-phenylindole (DAPI, Vector Laboratories), and examined under the microscope.

### Statistical analysis

Results are presented as a mean value ± standard deviation. P values were determined by a Mantel-Cox test and P values < 0.05 were deemed as statistically significant. Data were analyzed by GraphPad Prism, version 5.0.
